# Natural products that reduce rotavirus infectivity identified by a cell-based moderate-throughput screening assay

**DOI:** 10.1186/1743-422X-3-68

**Published:** 2006-09-01

**Authors:** Mark E Shaneyfelt, Anna D Burke, Joel W Graff, Mark A Jutila, Michele E Hardy

**Affiliations:** 1 Veterinary Molecular Biology, Montana State University, Bozeman, MT 59715, USA

## Abstract

**Background:**

There is widespread interest in the use of innate immune modulators as a defense strategy against infectious pathogens. Using rotavirus as a model system, we developed a cell-based, moderate-throughput screening (MTS) assay to identify compounds that reduce rotavirus infectivity in vitro, toward a long-term goal of discovering immunomodulatory agents that enhance innate responses to viral infection.

**Results:**

A natural product library consisting of 280 compounds was screened in the assay and 15 compounds that significantly reduced infectivity without cytotoxicity were identified. Time course analysis of four compounds with previously characterized effects on inflammatory gene expression inhibited replication with pre-treatment times as minimal as 2 hours. Two of these four compounds, α-mangostin and 18-β-glycyrrhetinic acid, activated NFκB and induced IL-8 secretion. The assay is adaptable to other virus systems, and amenable to full automation and adaptation to a high-throughput format.

**Conclusion:**

Identification of several compounds with known effects on inflammatory and antiviral gene expression that confer resistance to rotavirus infection in vitro suggests the assay is an appropriate platform for discovery of compounds with potential to amplify innate antiviral responses.

## Background

Interest in the use of innate immune modulating agents recently has increased in the context of developing effective biodefense strategies. Increasing natural disease resistance by administration of agonists that stimulate pathogen recognition receptors and gene expression pathways is an approach that would provide broad protection from infection without need for pathogen-specific vaccines. Stimulating broadly reactive immune responses to viral, as well as bacterial and protozoan pathogens, has shown efficacy in animal models and is the subject of recent reviews that address the utility of immune potentiators in developing infectious disease defense strategies [1,2]. The number of such compounds currently in clinical development lends support for this approach [3].

To be seriously considered as a candidate antiviral drug or innate immune agonist, rapid and quantitative assessment of activity and toxicity in cell culture are prerequisite to lead compound development [4]. Therefore, development of screening assays in cell lines that can simultaneously support virus replication and be responsive to measures of inhibition of virus replication are a priority. Cell-based high-throughput screening (HTS) assays that test for compounds active against hepatitis C virus (HCV), HIV and SARS coronavirus utilize recombinant viruses and changes in reporter gene expression in engineered cell lines to measure antiviral activity [5-8]. For example, a dual replicon assay system that combined reporter gene assays and FRET was used to screen for drugs effective against HCV [5]. Advantages of these cited assays include rapid readout and an ability to perform screens with viruses that must be handled under elevated biosafety conditions.

The intent of the studies reported here was to establish a cell-based screening assay that could identify compounds that inhibit virus replication by inducing antiviral gene expression pathways. Although the assay could identify compounds that target distinct steps of the virus replication cycle, we are interested in those that stimulate cellular responses necessary to confer initial resistance to rotavirus infection. The theory behind this approach is that amplification of the antiviral response will override virus-encoded immune evasion strategies and restrict replication to subclinical levels.

Rotaviruses are responsible for the majority of childhood morbidity and mortality from viral gastroenteritis [9]. Several rotavirus vaccines in clinical trials show promising efficacy, suggesting that a long-term goal of rotavirus gastroenteritis becoming a vaccine preventable disease is attainable [10]. However, the significant mortality associated with rotavirus illness in the developing world suggests approaches to enhance the antiviral immune response and consequent natural resistance to rotavirus infection need to be explored. The importance of this issue and the significant amount of data available on rotavirus replication led us to use this virus as a model system. We report development of an assay that uses unmodified adherent epithelial cells to measure reductions in rotavirus infectivity in response to treatment of cells with a variety of compounds. A natural product library consisting of 280 compounds derived from plant extracts was screened, and several compounds that inhibited rotavirus infectivity in a dose-dependent manner were identified. All of the compounds that passed the designated criteria of a hit have been reported to have direct effects on inflammatory or antiviral gene expression or on virus replication. Development of such a platform to screen compounds for the ability to diminish virus replication easily can be applied to other virus systems where direct measurement of activity in epithelial cells is desirable.

## Results

### Screening assay development

**Figure 1:**
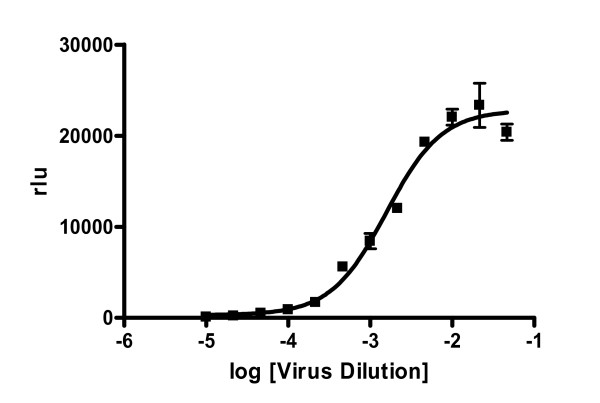
**Luminescent signal magnitude corresponds with infectious units**. Cells were infected with 10^-1/3 ^serial dilutions of rotavirus for 18 hours. Plates were fixed and probed with anti-VP6 mAb A6M followed by peroxidase-conjugated goat anti-mouse IgG. Signals were developed with chemiluminescent substrate and measured on a ThermoElectron Fluroskan and scored as relative light units (rlu). Error bars are standard errors of the means (n = 5).

An ELISA-based assay that successfully measured neutralization of rotavirus infectivity has been reported [11]. Similarly, we adapted an immunofluorescent (IF) infectivity assay to a moderate-throughput screening (MTS) format to measure changes in rotavirus infectivity following treatment of cells with compounds from a natural product library. In the standard IF assay, MA104 cells are cultured to confluence in 96-well microtiter plates, then infected with rotavirus in triplicate wells and incubated for 18–20 hours. Virus replication then is detected by indirect IF and replication is quantified by counting fluorescent focus forming units (FFU) in a dilution series by fluorescence microscopy. To adapt the assay to an MTS format that would necessarily eliminate manual counting of FFU, an HRP-conjugated secondary antibody was used in place of the FITC-conjugated antibody, and the assay was developed with chemiluminescent substrate. Virus replication then was quantified in a microplate fluorometer with readout of relative light units (rlu). Use of an enzymatic signal to measure infectivity was validated by comparing changes in chemiluminescent signal versus virus dose measured by IF. The data shown in figure 1 illustrate the range of increase in chemiluminescent signal that corresponds with increasing infectious units in a 10^-1/3 ^virus dilution series. These data demonstrate that changes in the magnitude of the enzymatic signal accurately reflect changes in the number of infectious units measured by IF.

**Figure 2:**
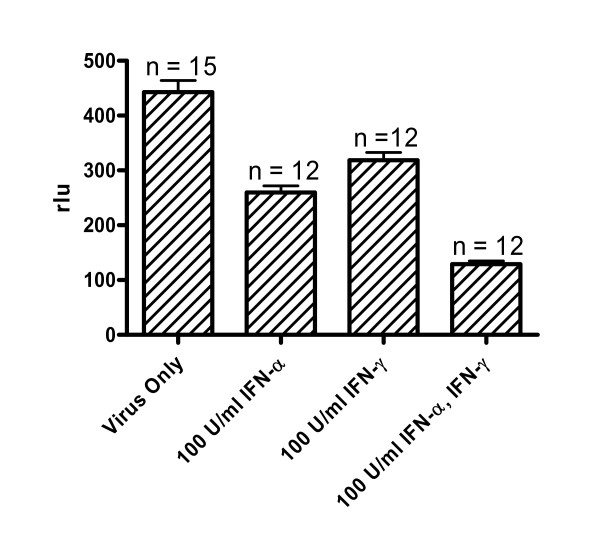
**Establishment of internal controls: IFNα and IFNγ reduce rotavirus infectivity in the assay**. Cells were treated with indicated amounts of each cytokine or a combination of both prior to infection. Infections were allowed to proceed for 18 hours and chemiluminescent measurement of reduction in rotavirus infectivity was performed as described in the text and in the legend to Figure 1. Error bars are standard errors of the means.

In order to establish internal inhibition controls for the assay, we measured reductions in infectivity in response to cytokines known to induce antiviral gene expression. Cells were pre-treated with IFNα, IFNγ, or a combination of both cytokines. IFNα and IFNγ both reduced infectivity, and the magnitude of reduction increased to ~80% when cells were treated with the combination of both cytokines (Figure 2). The combination of IFNα and IFNγ consistently yielded the greatest reduction in virus replication. Therefore, this mixture of cytokines, and IL-2 which is not known to have direct antiviral activity, were included as internal positive and negative controls, respectively, in each plate of every experiment.

### Assay validation

**Figure 3:**
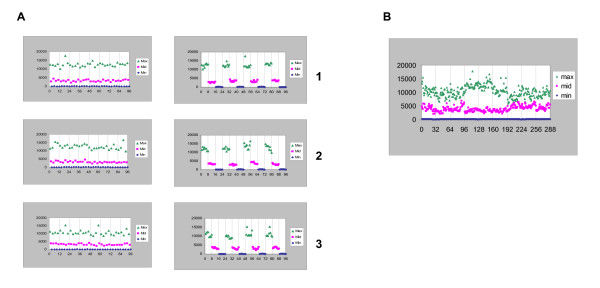
**Assay validation**. Cells were treated according the provided template [14]. Three individual experiments, each containing three plates, were conducted on three separate days. A) The left panels show a representative set of data from day two of the plate uniformity assay with the data graphed in a well-row orientation where the wells are labeled horizontally. The panels on the right contain the same data in a well-column orientation. B) Aggregate data of all nine plates over three days in a well-row orientation.

Plate uniformity assessments were performed according to the recommendations of the NIH Chemical Genomics Center in order to determine whether our assay was suitable for eventual adaptation to a high-throughput format. The Z' coefficient was established with mock infected cells representing the minimum signal value, virus infected cells representing the maximum signal value, and the IFNα/IFNγ mixture as the midrange signal. Experiments consisting of three plates each run on three consecutive days were performed and an example of the data from one day is shown in Figure 3. No evidence of significant drift or edge effect was observed. The Z' coefficient measures the quality of an HTS assay by comparing data variation and the signal dynamic range, and a value of > 0.5 establishes the assay as excellent for screening [12,13]. The Z' values ranged from 0.69 – 0.82 for individual plates, and the aggregate Z' value from all plates and all days was 0.64. A signal-to-noise value of 4.96 was calculated with the aggregate data from all plates. All of the statistical criteria for intra-plate assessment were met [14].

The assay results show some inter-plate variability. For example, although all within-day fold-shifts in signal were less than 2, all average (between)-day fold-shifts were not. We expect some degree of variability due simply to the variations in the growth status of cells from day to day. Importantly, we established sufficient intra-plate controls to account for minor variances associated with cell status. Together the validation and statistical data support the assay as a viable platform for screening compound libraries.

### Compounds from a natural product library reduce rotavirus infectivity

**Figure 4:**
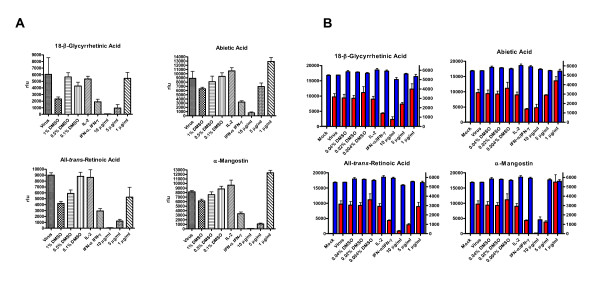
**Natural products identified in the MTS assay reduce rotavirus infectivity in a dose-dependent manner**. Representative data are shown for four compounds: 18-β-glycyrrhetinic acid, abietic acid, mangostin, and all-*trans *retinoic acid. A) Data from initial screen. The *y*-axis is relative light units (n = 3). B) Secondary screen that includes a dose-response (red) and cell viability (blue) data. The left *y*-axis is relative light units and the right *y*-axis is viability (n = 3). Error bars are standard errors of the means. The highest concentration of mangostin was toxic under the conditions of this assay.

A 280 compound natural product library was screened to identify compounds that reduced rotavirus infectivity. Forty-seven (17%) compounds were selected for a second round of screening and were tested both for dose-dependency and for effects on cell viability. A representative dataset is shown in Figure 4. Figure 4A shows data obtained from the initial screen, and the data in figure 4B illustrate dose-dependent reductions in rotavirus infectivity in response to treatment of cells with each of the four representative compounds. The observed reduction in infectivity was not a result of generalized cytotoxicity because cell viability did not decrease upon treatment beyond the DMSO only controls (Figure 4B). The one exception was that mangostin was toxic at 10 μg/ml. Dose-dependency and cell viability assays were performed in parallel with cells seeded at the same densities from the same cell suspension.

Forty-seven compounds from the primary screen were subjected to second round screening and 32 were eliminated from consideration for follow-up studies. Ten compounds did not reduce infectivity above the established threshold in the second round, generating a true false positive hit rate of ~20%. Twenty compounds proved cytotoxic as measured by the cell viability assay, and two compounds are known toxins with gross effects on cell metabolism (e.g. protein synthesis inhibitors). The two toxins were eliminated from further consideration because we are interested in identifying compounds that stimulate or enhance antiviral signaling pathways. The remaining 15 compounds were designated true hits (5 %) and are listed in Table 1.

**Table 1:** Compounds that reduce rotavirus infectivity without cytotoxicity

Manufacturer's ID Number	Chemical Name	FW	Chemical Family	Known Functions (Select references)
**Group A**				
TNP00130	18-β-Glycyrrhetinic Acid	470.68	Triterpene	-Antiviral activity against a number of DNA and RNA viruses [25] -Inhibits gap junction communication
				
TNP00088	Abietic Acid	302.45	Diterpene	-Inhibits acute inflammation after topical or oral administration [26] -Reduces neutrophil infiltration [26] -Reduces COX-2 and TNF-α expression [16] -Activates PPAR-γ in macrophages [16]
				
TNP00194	All-*trans*-Retinoic Acid	300.44	Retinoid	-Increases expression of type I interferon receptors [27]
				
TNP00140	α-Mangostin	410.46	Xanthone	-Used in treatment of skin infections, wounds and diarrhea in Southeast Asia [15] -γ-Mangostin inhibits NF-κB activation and COX-2 expression [15] -α-Mangostin preferentially inhibits growth of HL60 cells [28] -Induces caspase-9 and -3 activation in HL60 cells [28]
				
**Group B**				
TNP00307	Kinetin-9-Riboside	347.33	Phytohormone	-Cytokinin -Antiviral activity against Tobacco Mosaic Virus [29] -Causes a decrease in (poly rI) (poly rC) stimulated interferon response in VSV challenged mice [30] -Reverses the effect of endotoxin-enhanced host resistance [31]
				
TNP00064	7,3'-Dihydroxyflavone	254.24	Flavone	
				
TNP00050	6,7-Dimethoxyflavone	282.29	Flavone	
				
TNP00044	8-Hydroxy-7-Methoxyflavone	268.27	Flavone	
TNP00151	Genistein	270.24	Isoflavone	-Tyrosine kinase inhibitor [32] -Down-regulates iNOS [33] -Inhibits NF-κB activation [33] -Inhibits COX-2 induction [33] -Inhibits LPS induced IL-1β, IL-6, and TNF-α production in monocytes
				
TNP00227	Capsaicin	305.42	Phenylalkyl-Amine Alkaloid	-Inhibits NF-κB activation [34] -Activates transient receptor potential vanilloid-1 (TRPV-1) [35]
				
TNP00256	Securinine	217.26	Pseudoalkaloid	-GABA receptor antagonist -Antibacterial activity against E. coli, Staph. aureus and Myc. smegmatis [36] -Antimalarial activity [37]
TNP00231	Isopimaric Acid	302.46	Diterpene	-Inhibitory activity against multidrug-resistant strains of *Staphylococcus aureus *[38] -activates large-conductance Ca^2+^-activated K^+ ^channel α-subunit [39]
TNP00292	Parthenolide	248.32	Sesquiterpene Lactone	-inhibits NFκB activation by preventing induction of IκB kinase [40]
				
TNP00006	Unknown	358.48		
TNP00014	Unknown	388.50		

### Time course of inhibitory effects

**Figure 5:**
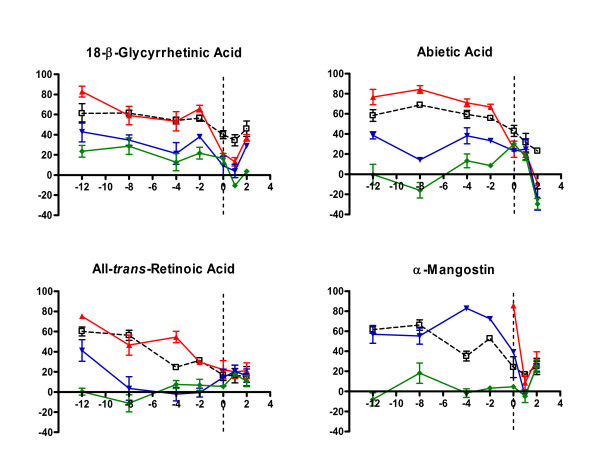
**Time course of inhibition of rotavirus replication**. Cells were treated with indicated compounds for various intervals ranging from 12 hr pre-infection to 2 hr post-infection. Each time point was assayed in triplicate and virus was quantified by counting FFU. Open squares are IFN control. Red, 7.5 μg/ml, blue, 5 μg/ml, and green, 2.5 μg/ml The *y*-axis represents percent inhibition and the *x*-axis is time of infection. Error bars are standard errors of the means (n = 3).

The four compounds shown as representative data in Figure 4 were analyzed further because of prior reports of their effects on components of innate immune signaling pathways [15-17]. A time-course and dose-response of each compound were established in the immunofluorescent focus reduction assay. Cells were treated with each compound for various times ranging from 12 hours pre-infection to 2 hours post-infection (Figure 5). Significant levels of inhibition of virus replication were observed with each compound when added as early as 2 hours prior to infection. The level of inhibition did not change dramatically as pre-treatment times increased up to 12 hours. All compounds showed some ability to reduce infectivity when added at the time of infection, and 18-β glycyrrhetinic acid, all-*trans *retinoic acid and mangostin were somewhat effective when added at 1 or 2 hours post-infection. Interestingly, addition of abietic acid post-infection showed a small, but significant increase in infectivity that also was dose-dependent.

### α-mangostin and 18 β-glycyrrhetinic acid activate NFκB and induce IL-8 secretion

**Figure 6:**
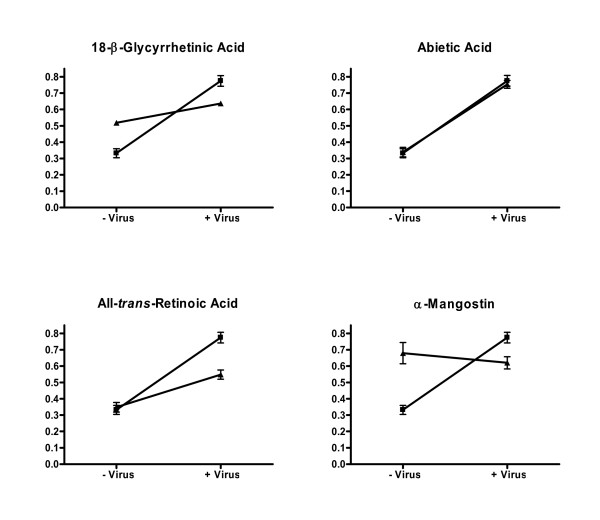
**NFκB activation in infected and uninfected cells in the presence of selected compounds**. Infected or mock infected MA104 cells were treated with indicated compounds at a final concentration of 7.5 μg/ml. Nuclear extracts were prepared 6 hours post-infection and activation of NFκB was assessed and quantified by commercial ELISA. Squares indicate DMSO only treated cultures and triangles indicate compound treated cultures. The *y*-axis is OD_450 _nm. The data were analyzed by two-way ANOVA (n = 3).

**Figure 7:**
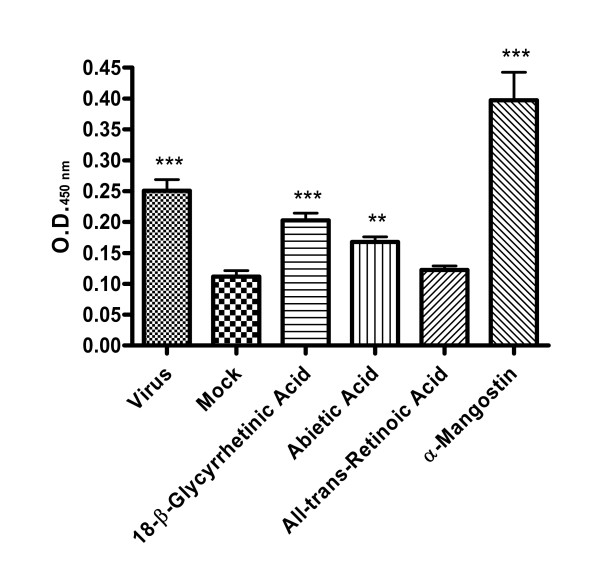
**IL-8 expression cells treated with selected compounds**. MA104 cells were treated with indicated compounds for 24 hours and IL-8 in the supernatants was measured with a commercial ELISA. ** = p < 0.01, *** = p < 0.001, n = 4.

The design of our assay measures inhibition of rotavirus replication but does not distinguish whether antiviral signaling pathways are activated or whether the compounds block specific steps of the virus replication cycle. To test the hypothesis that virus replication was reduced because cell signaling pathways involved in antiviral and inflammatory gene expression were induced by selected compound treatment, NFκB activation and IL-8 secretion was measured following each treatment. α-mangostin and 18 β-glycyrrhetinic acid induced NFκB activation, whereas all-*trans *retinoic acid and abietic acid did not beyond the levels of the control (Figure 6). The levels of IL-8 expression measured for each compound were consistent with levels of activation of NFκB (Figure 7).

## Discussion

We designed a MTS assay capable of identifying compounds that reduce rotavirus infectivity, toward a long-term goal of discovering compounds that activate innate immune signaling pathways to reduce the disease impact of acute viral infections. We screened a library consisting of 280 natural products purified from plant extracts, and several compounds were identified that reproducibly inhibited rotavirus replication without cytotoxicity. The assay has been validated statistically, as well as by the observation that compounds selected for further study were purchased from different sources and showed reproducible inhibition of virus replication. In addition, follow-up assays measured reductions in infectivity in response to compound treatment in the standard rotavirus IF assay and the degrees of replication inhibition and dose-dependence correlated with the values obtained in the MTS assay. We calculated a true hit rate for the natural product library of 5% and a false positive rate of ~20%. We performed numerous optimization assays to reduce variability presented by variation in growth properties of our cell line. We also have initiated a screen of a 10,000 compound synthetic chemical library, and after screening ~2500 compounds, our true hit rate is 1.8% (data not shown). These true hit percentages yield a large, but not prohibitive, number of compounds for follow-up studies both in vitro and in animal models. Together the data suggest our assay is an effective platform for screening candidate compounds for antiviral activity in adherent epithelial cells.

In this study, we sought to identify compounds with innate immune modulating effects that resulted in cellular resistance to rotavirus infection. The format of the assay, that is, readout of reduction in infectivity, does not distinguish between compounds that act on antiviral signaling pathways and those that may target specific steps in the virus replication cycle, such as entry or replicase activity. However, two of the four compounds selected for follow-up study activated NFκB and induced IL-8 secretion. Moreover, *the majority of compounds called a hit in the screen for which data is available has been previously described to affect inflammatory or anti-inflammatory gene expression or pathogen growth in vitro *(Table 1). These observations strongly support the assertion that our assay is appropriate, but not exclusive, for discovery of immune potentiators, as we intended.

There has been a resurgence of interest in natural products as drug candidates for a variety of reasons including increased interest in infectious disease prevention and therapy, a lower percentage of chemical properties that negatively affect permeation and absorption, and the propensity of natural products to act by affecting protein-protein interactions [18]. Important for the studies described here, natural products are known to modulate immune responses and cell signaling pathways. Table 1 lists compounds designated hits in this study, along with some of the reported functions. Compounds were grouped into those chosen for further study (Group A), and those known to stimulate or repress inflammatory responses or innate immune signaling pathways and two with unknown functions (Group B). Also included in Group B are compounds with reported antiviral, antibacterial, or anti-protozoan activity. We have not yet deciphered mechanisms by which these compounds inhibit rotavirus replication in vitro. However, the fact that several of the compounds, for example, abietic acid, genistein, and capsaicin, interfere with NFκB activation and cyclo-oxygenase 2 (COX2) expression is noteworthy (see Table 1 for references). NFκB activation is an important regulator of COX2 expression; COX2 activity and COX-mediated prostaglandin synthesis is necessary for rotavirus infectivity in CaCo-2 intestinal cells [19]. Interestingly, the addition of prostaglandin E_2 _(PGE_2_) restored infectivity reduced by the COX inhibitor [19] and three of the compounds we chose for follow-up studies all have an inhibitory effect on either synthesis or release of PGE_2 _(see Table 1 references). The ability of α-mangostin and 18 β-glycyrrhetinic acid to inhibit rotavirus replication when both compounds activate NFκB is most likely because antiviral states are established upon treatment of the cells with each compound prior to infection. The definitive mechanisms by which the compounds identified in this natural product library screen warrant further investigation.

We intend this MTS assay to serve as a platform for discovery of candidate adjuvants that will be effective against acute viral infections at mucosal surfaces. Rotavirus is an ideal model system for these purposes for several reasons. First, rotaviruses cause gastrointestinal illness in most mammalian species and so their relevance as mucosal pathogens is clear. Second, these viruses are well characterized with respect to structure, antigenicity, and mechanisms of virus replication, and thus an excellent resource for mechanistic follow-up studies is available. Third, rotavirus is promiscuous in its tropism for cultured cell lines, and multiple cell lines of different types and species of origin, including primary cell lines [20], support productive virus replication. The ability to propagate rotavirus in a variety of cell types supports high throughput applications that study general as well as cell-type specific innate immune responses. Fourth, both small and large animal models of natural infection allow relatively rapid evaluation of the efficacy of candidate compounds in vivo. Finally, the fact that rotavirus employs a mechanism to down-regulate antiviral gene expression allows consideration of possible evasion strategies when selecting and testing candidate immune potentiators [21,22]. Current development efforts include expansion of screening studies to other lytic RNA viruses such as influenza virus, and adaptation of the assay to a fully automated format.

## Methods

### Cells, virus, and rotavirus monoclonal antibodies

MA104 monkey kidney epithelial cells were maintained in M199 medium (MediaTech Cell Grow) supplemented with 5% fetal bovine serum (FBS; Atlanta Biologicals). Isolation and cultivation of G serotype 6 (G6) bovine rotavirus strain NCDV has been described [23]. Monoclonal antibody (MAb) E4 reacts with major structural protein VP6 of most group A rotavirus strains [24]. MAb A6M recognizes VP6 and was generated by immunizing mice with G6 bovine rotavirus strain B641 and screening hybridoma supernatants for antibodies that react with rotavirus specific proteins.

### Reagents and chemicals

A library containing 0.5 mg each of 280 natural products purified from plant extracts was purchased from TimTec (TimTec Corporation). Compounds were reconstituted in 500 μl of dimethyl sulfoxide (DMSO) and the library was stored at -80°C. Individual compounds α-mangostin (TimTec or Indofine), 18-β-glycyrrhetinic acid (18-BGA; Fluka), abietic acid (AA; Sigma), and all-*trans*-retinoic acid (ATRA; Sigma) were reconstituted in DMSO to final stock concentrations of 25 mg/ml.

Recombinant human interferon-α (IFN-α; BioSource International, Inc) was diluted to a concentration of 1 × 10^5 ^U/ml in phosphate buffered saline (PBS) containing 0.1% bovine serum albumin (BSA). Recombinant human interferon-γ (IFN-γ) and recombinant human interleukin-2 (IL-2; Peprotech Inc) were diluted to 2 × 10^6 ^U/ml and 10 μg/ml, respectively, in PBS.

Horseradish peroxidase (HRP)-conjugated goat anti-mouse IgG (H+L), HRP-conjugated goat anti-mouse IgG (H+L) F(ab')_2 _fragments, and FITC-conjugated goat anti-mouse IgG were purchased from Jackson ImmunoResearch Laboratories.

### Screening assay

#### Compound treatments and virus infections

Working stock solutions of each compound from the library were prepared to twice the desired final concentrations of 20 μg/ml, 10 μg/ml, and 2 μg/ml in serum-free M199. Working stock solutions of assay controls consisted of 2%, 1% DMSO, and 0.2% DMSO, a mixture of 200 U/ml each of IFN-α and IFN-γ, and 2 ng/ml of IL-2.

MA104 cells were cultured to confluence in 96-well black-walled plates (Costar). The culture media was decanted and replaced with 50 μl of M199. 50 μl of 2X control and experimental stock solutions were added to respective wells, in triplicate, and plates were incubated for 4 hours at 37°C. Following the 4 hour incubation, the contents of each plate were removed and 8.9 × 10^5 ^ffu/well of trypsin-activated NCDV in 0% M199 was added to appropriate wells. Mock infected wells received 50 μl of 0% M199. 50 μl of fresh 2X control and experimental compounds were added and at 18 hours post-infection, the cells were fixed for 10 minutes with 80% acetone.

#### Cell-based ELISA

The wells were blocked for one hour at room temperature with 100 μl of PBS containing 3% bovine serum albumin (w/v) and 0.05% Tween-20. The plates were washed one time with 400 μl of wash buffer consisting of PBS and 0.05% Tween 20. 50 μl of 20 μg/ml A6M or E4 (1:25 hybridoma supernatant) in PBS containing 0.05% Tween 20 and 0.5% dry milk was added to the wells and incubated for one hour at room temperature. The plates were washed four times with wash buffer, then 50 μl of a 1:500 dilution of HRP-conjugated F(ab')_2 _in 0.5% Blotto was added and the plates were incubated for one hour at room temperature. Following a final wash, 100 μl of BM Chemiluminescence ELISA Substrate (Roche Diagnostics) was added and reactions were allowed to proceed for 4 minutes to reach a steady state of enzymatic activity. Signals were measured on a ThermoElectron Fluroskan (ThermoElectron Cooperation) with an integration time of 1,000 ms.

### Cell viability assay

Cell viability assays for compound toxicity were set up similar to compound screening except cells were not infected over the course of the experiments. Cell viability was measured with the CellTiter-Glo Luminescent Cell Viability Assay (Promega) according to instructions provided by the manufacturer.

### Statistics and criteria for "hit" designation

Compounds that showed a greater than 60% decrease in signal at 5 μg/ml when compared to the 0.5% DMSO control, and p < 0.05 as determined by a one-tailed student's *t *test, were selected for secondary screening. Additionally, compounds that showed a greater than 90% decrease in signal when compared to the 1% DMSO control and p < 0.05 also were selected for further screening. Compounds that showed a greater than 10% decrease in signal and a p < 0.05 in the cell viability assay were determined to be toxic.

### Plate uniformity assessment

The recommendations of the National Institutes of Health Chemical Genomics Center's *Assay Guidance Manual Version 4.1 *for plate uniformity assessments were followed. Three separate experiments consisting of three plates each were performed on three different days. Confluent MA104 cells were pretreated for 4 hours with 100 μl of either media or a mixture of 100 U/ml each of IFN-α and IFN-γ. All media was serum free and contained 1% DMSO.

The pre-treatment media was decanted and the cells were infected with 50 μl of 8.9 × 10^5 ^pfu/ml of trypsin-activated NCDV. Mock infected wells were treated with 50 μl of serum free media. 50 μl of fresh 2X treatment media containing 2% DMSO was added to the respective wells and the plates were incubated for 18 hours. The plates were fixed, labeled and quantified according to the assay procedure described above. All calculations were performed using the Assay Guidance Manual's spreadsheet [14].

### Immunofluorescent focus assay

Immunofluorescent assays (IF) for rotavirus infectivity were performed as previously described [24]. Cells were cultured in 96-well plates and were mock infected or infected with NCDV at approximately 150 ffu/well. Pre- or post-treatment with compounds was performed for the indicated times as described above. 18 hours post-infection, the cells were fixed for 10 minutes with 80% acetone. Incubations with primary and secondary antibody were as described above, except the secondary antibody was FITC-conjugated goat anti-mouse IgG. Fluorescent foci were counted by microscopy (Nikon Eclipse TE300).

### NFκB and IL-8 assays

MA104 cells in 100 × 20 mm culture dishes were treated with 7.5 μg/ml of selected compounds in serum-free M199 containing 0.03% DMSO. The effects of the presence of virus on NFκB activation and IL-8 secretion in the assays was tested by infecting cells with trypsin-activated NCDV at an moi of 10 pfu/cell at the time of compound treatment. In all cases, incubation periods were 6 hours.

NF-κB activation was quantified with TransAM NF-κB p50 Transcription Factor Assay Kit (Active Motif). Nuclear extracts were prepared with CelLytic NuCLEAR Extraction Kit (Sigma) following the manufacturer's protocol. Nuclear protein concentration was determined with the D_c _Protein Assay (Bio-Rad Laboratories), and 20 μg of nuclear protein was used in the assay. Statistical analysis of the data was performed by two-factor ANOVA.

IL-8 secretion was measured with the Quantikine Human IL-8 system (R & D Systems) following the manufacturer's protocol. Compound treatments and infections were performed as described above, except that the incubation periods were extended to 24 hours. The data were analyzed by student's *t *test with a threshold of significance set to *p *< 0.001.

## Competing interests

The author(s) declare that they have no competing interests.

## Authors' contributions

MES established the assay, performed most of the experiments and assisted in preparation of the manuscript. ADB performed a significant number of screening assays and data analysis, and participated in manuscript preparation. JWG purified and characterized virus stocks, assisted in assay development and in manuscript preparation. MAJ contributed to study design, assay development, data analysis and manuscript preparation. MEH conceived of the study, participated in design and is responsible for study oversight.
